# Neural Remodeling of the Left Atrium in Rats by Rosuvastatin Following Acute Myocardial Infarction

**DOI:** 10.1515/biol-2019-0068

**Published:** 2019-12-31

**Authors:** Jiang-Rong Wang, Meng-Zan Wang, Shao-Hua Zheng, Zhi-Yuan Li

**Affiliations:** 1 Department of Cardiology, The First Affiliated Hospital of Shandong First Medical University, Jinan, Shandong 250014, China; 2 Department of Cardiology, People’s Hospital of Liaocheng, Liaocheng, Shandong 252000, China; 3 Department of Cardiology, People’s Hospital of Dongying, Dongying, Shandong 25700, China

**Keywords:** rosuvastatin, myocardial infarction, atrial remodeling, arrhythmia, neural remodeling

## Abstract

**Objective:**

This study aims to investigate the effect of rosuvastatin on sympathetic neural remodeling of the left atrium (LA) in rats after myocardial infarction (MI).

**Methods:**

Rats were randomly divided into a three groups: sham group, statin group, and MI group. The mRNA expression levels of the growth-associated protein-43 (GAP43) and nerve growth factor (NGF) were measured by RT-PCR. Immunohistochemistry was used to detect the distribution and density of GAP43- and NGF-positive nerves. The expression levels of these proteins were quantified by Western blot.

**Results:**

Compared with the sham group, the average optical density (AOD) values of GAP43 and nerve growth factor (NGF)-positive substances in the LA in the statin and MI groups were significantly higher (*P*<0.01), but the AOD values in the statin group were lower than of those in the MI group (*P*<0.01). Furthermore, the AOD values of GAP43 and NGF positive nerves in the left stellate ganglion in the statin and MI groups were significantly higher (*P*<0.01), but the AOD values in the statin group were lower, when compared with the MI group (*P*<0.01).

**Conclusion:**

Rosuvastatin could effectively improve the sympathetic neural remodeling of LA in MI rats.

## 1 Introduction

The heart is innervated by intrinsic and extrinsic autonomic nerves. The stellate ganglion (SG) is formed by the fusion of the inferior cervical ganglion and the first thoracic ganglion. Sometimes, it also includes the second thoracic ganglion. Functionally, it is a sympathetic ganglion. The left stellate ganglion (LSG) mainly innervates the left side of the heart and the majority of left and right ventricles. Enhanced excitability of that side of the ganglion increases the susceptibility to ventricular arrhythmias [1]. After the occurrence of myocardial infarction (MI), the phenomena of neural remodeling would appear, including denervation, nerve sprouting, and excessive degeneration [2]. Studies [3, 4] have shown that cardiac neural remodeling after MI could promote atrial tachyarrhythmias, especially the occurrence of atrial fibrillation (AF). In recent years, the pleiotropic effects of statins have received considerable attention. In addition to regulating blood lipids, anti-arrhythmia functions and the prevention of cardiac remodeling have been frequently reported [5, 6]. A large clinical trial conducted by Cao *et al*. [7] already confirmed that statins have anti-arrhythmia functions. However, the specific mechanism remains unclear. The present study aimed to investigate whether intrinsic and extrinsic autonomic neural remodeling in the heart occurred after MI, and examines the effect of rosuvastatin on atrial neural remodeling in rats after MI and its possible mechanism.

## 2 Material and Methods

### 2.1 Establishment of animal models and grouping

Male Wistar rats, weighing 220-260 g (purchased from Experimental Animal Center, Affiliated Hospital of Shandong University of Traditional Chinese Medicine, Jinan, China), were studied. All rats were housed for a two-week acclimatization period before the experiments were conducted. Rats were randomly assigned to the following experimental groups: (1) myocardial infarction group (MI group, *n*=17); (2) MI+rosuvastatin group (statin group, *n*=19); and (3) sham control group (sham group, *n*=21). The MI model was established in the MI and statin groups. After intraperitoneal anesthesia, a thoracotomy was conducted to expose the heart. The anterior descending artery was ligated using a 6-0 suture. After the formation of MI was confirmed by the observation of white distal myocardia and left atrial filling, the thorax was closed layer by layer. In the sham group, the same location was sutured, but not ligated. After MI, rats in the statin group were intragastrically administered with 0.8 6mg/(kg·d) of rosuvastatin (AstraZeneca, Wuxi, China), while the sham group and MI group were given equal volume of normal saline for four weeks.

**Ethical approval**: The research related to animals use has been complied with ‘The Guide for the Care of Use of Laboratory Animals’ published by the National Institutes of Health (NIH, Publication No. 85-23, revised 1996) and was approved by the Animal Care Committee of the Medical College of Shandong University.

### 2.2 Collection of myocardial tissue specimens

Four weeks after surgery, rats in each group were sacrificed, and the hearts were rapidly removed and washed with a 0.9% NaCl solution. The LSG and myocardia were collected from the left atrium (LA) at approximately 0.4-0.6 cm^2^. Part of the tissues were fixed in neutral formalin for one hour and stored in 4% formalin. Then, parts of the tissues were collected and stored in liquid nitrogen for Western blot and reverse transcription polymerase chain reaction (RT-PCR) detection.

### 2.3 Immunohistochemistry

The sample sectioning procedures and details of the staining techniques were performed as previously described. In order to detect nerve fibers in the heart, antibodies against nerve growth factor (NGF, AB52918, 1:1,000; Abcam, Cambridge, UK) and growth associated protein 43 (GAP43; AB75810, 1:1,000; Abcam) were used. An image processing system was used to quantitatively detect the NGF- and GAP43-positive regions through the use of immunohistochemical stains, and these values were expressed as the average optical density (AOD).

A computer-assisted morphometric analysis system (Image-Pro Plus 6.0) was used to determine nerve density. The computer automatically recognized the color of the immunohistochemical stains and calculated the pixel area of the nerves. Each slice was observed at 40× under a microscope. The image was saved at a resolution of 4080×3072 pixels. One to three sections from each animal were observed, and three maximum and minimum nerve density fields from each section were selected. The average density of the maximum nerve density fields was taken as the average density of a section. The average difference between the maximum and minimum nerve density fields was defined as the heterogeneity of the local nerve distribution.

### 2.4 MRNA analysis

Total RNA was extracted from the LA and the LSG using TRIzol reagent, according to the manufacturer’s protocol. Then, this was treated with DNase I to degrade any trace of DNA, and purified using an RNeasy Kit (Takara, Tokyo, Japan). The integrity of each sample was confirmed by agarose gel analysis. Total RNA was reverse transcribed using TaqMan reverse transcription reagents (Takara). The expression levels of candidate genes were measured by real-time quantitative RT-PCR (qRT-PCR) using Power SYBR Green (Takara) dye, and quantified using ViiA7 (Applied Biosystems, CA, USA). In each assay, both an endogenous control gene (β-actin) and a target gene from the same samples were amplified in duplicate in separate tubes. The mRNA levels of each target gene were calculated using the relative standard curve method and normalized against the corresponding β-actin mRNA levels. The expected amplicon sizes were confirmed by gel electrophoresis. The sequences of the genes studied were obtained from GenBank, and the primers were designed using Primer Premier 5.0 software (Premier Biosoft). Table 1 presents the primer sequences and amplicon sizes of the selected genes.

**Table 1:** mRNA primer sequences

Gene name		Primer sequence	Amplicon Size (bp)
β-actin	sense antisense	5’TCGTGCGTGACATTAAAGAG 3 ’ 5’CCAGGATAGAGCCACCAAT 3’	417 bp
Gap43	sense antisense	5’ GAGGAGCCTAAACAAGCCGA 3 ’ 5’ TGAGCAGGACAGGAGAGGAA 3’	319 bp
NGF	sense antisense	5’ GCGTTTTTGATCGGCGTACA 3 ’ 5’ CTCCAACCCACACACTGACA 3’	403 bp

bp, base pair; GAP43, growth-associated protein-43; NGF, nerve growth factor.

### 2.5 Western blot

Equal amounts of protein (50 μg) were separated on 8–16% Tris-glycine gels (Novex, Invitrogen, CA, USA) and transferred onto polyvinylidene fluoride (PVDF) membranes. After blocking with 5% skim milk, the PVDF membranes were incubated overnight at 4°C with the following primary antibodies: rabbit polyclonal anti- NGF antibody (AB52918, 1:1,000; Abcam), anti-GAP43 antibody (AB75810, 1:1,000; Abcam) and mouse monoclonal anti-GAPDH antibody (1:2,000; Abcam). On the next day, the membranes were incubated with horseradish peroxidase-conjugated secondary antibodies with appropriate species specificity. Immunoreactivity was visualized using an enhanced chemiluminescence (ECL) detection kit (LAS-4000 mini, Fujifilm, Japan). The immunoreactive bands were quantified by densitometry using the LAS-4000 mini 2200 software (Fujifilm) to estimate the protein expression. All protein expression signals were normalized to the intrasample GAPDH signal.

### 2.6 Statistical analysis

Data analysis and processing were performed using the SPSS 16.0 software. Quantitative data were presented asx ± standard deviation (SD). Comparison among multiple groups was performed using one-way analysis of variance (ANOVA). Pairwise comparison among multiple groups was performed using the least significant difference (LSD) test.

## 3 Results

### 3.1 Regeneration of the sympathetic nerve in the LA in each group after four weeks

**Figure 1:**
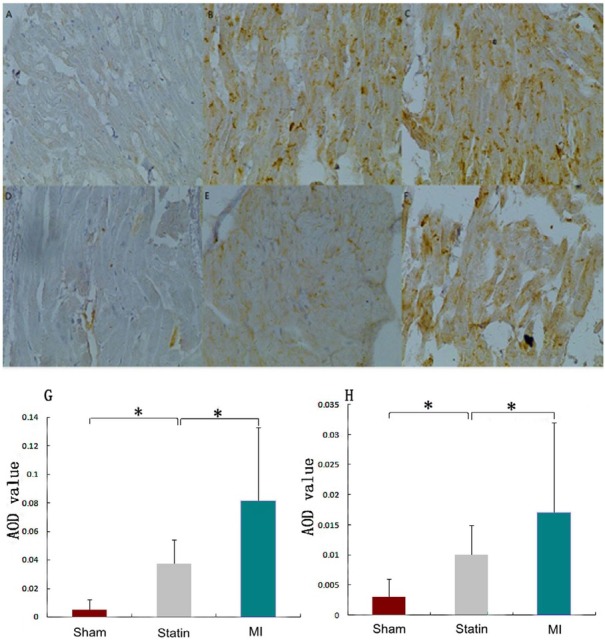
GAP43 and NGF positive nerve fibers in the LA in each group A: Sham group, GAP43 stain; B: Statin group, GAP43 stain; C: MI group, GAP43 stain; D: Sham group, NGF stain; E: Statin group, NGF stain; F: MI group, NGF stain; G: AOD values of GAP43 positive substances in rat atrium in the 3 groups; H: AOD values of NGF positive substances in rat atrium in the 3 groups. AOD: average optic density. The slides are shown at a magnification of 40. *: P < 0.01.

Compared with the sham group, the AOD values of GAP43 positive substances in the LA significantly increased in the statin and MI groups (0.037 ± 0.017 and 0.081 ± 0.052 *vs*. 0.005 ± 0.007, *P*<0.01). Compared with the MI group, the AOD value of GAP43 positive substances significantly decreased in the statin group (0.037 ± 0.017 *vs*. 0.081 ± 0.052, *P*<0.01). Compared with the sham group, the AOD values of NGF positive substances in the LA significantly increased in both the statin and MI groups (0.010 ± 0.005 and 0.017 ± 0.015 *vs*. 0.003 ± 0.003, *P*<0.01). Compared with the MI group, the AOD values of NGF positive substances significantly decreased in the statin group (0.01 ± 0.005 *vs*. 0.017 ± 0.015, *P*<0.01; Figure 1).

### 3.2 Regeneration of sympathetic nerves in the LSG in each group after four weeks

**Figure 2:**
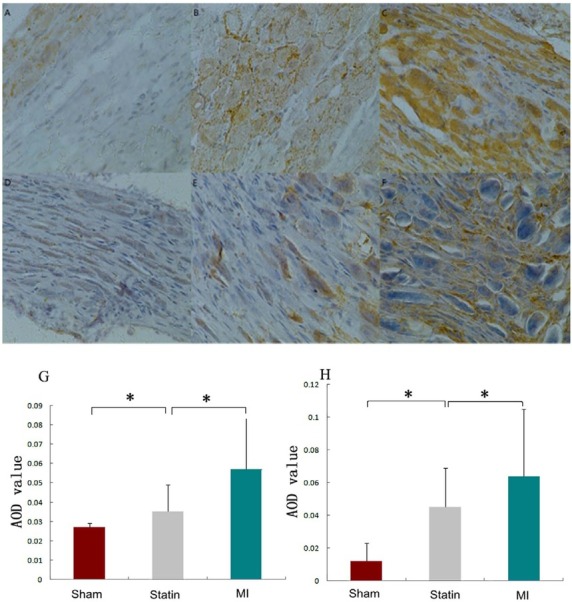
GAP43 and NGF positive nerve fibers in rat LSG in each group A: Sham group, GAP43 stain; B: Statin group, GAP43 stain; C: MI group, GAP43 stain; D: Sham group, NGF stain; E: Statin group, NGF stain; F: MI group, NGF stain; G: AOD values of GAP43 positive substances in rat LSG in the 3 groups; H: AOD values of NGF positive substances in rat LSG in the 3 groups. AOD: average optic density. The slides are shown at a magnification of 40. *: P < 0.01.

Compared with the sham group, the AOD values of GAP43 positive substances in the LSG significantly increased in both the statin and MI groups (0.035 ± 0.014 and 0.057 ± 0.027 *vs*. 0.027 ± 0.002, *P*<0.01). Compared with the MI group, the AOD values of GAP43 positive substances in the LSG significantly decreased in the statin group (0.035 ± 0.014 *vs*. 0.057 ± 0.027, *P*<0.01). Compared with the sham group, the AOD values of NGF positive substances in the LSG significantly increased in both the statin and MI groups (0.045 ± 0.024 and 0.064 ± 0.041 *vs*. 0.012 ± 0.011, *P*<0.01). Compared with the MI group, the AOD values of NGF (0.045 ± 0.024 *vs*. 0.064 ± 0.04, *P*<0.01) positive substances in the LSG significantly decreased in the statin group (*P*<0.01) (Figure 2).

### 3.3 MRNA of GAP43 and NGF

**Figure 3:**
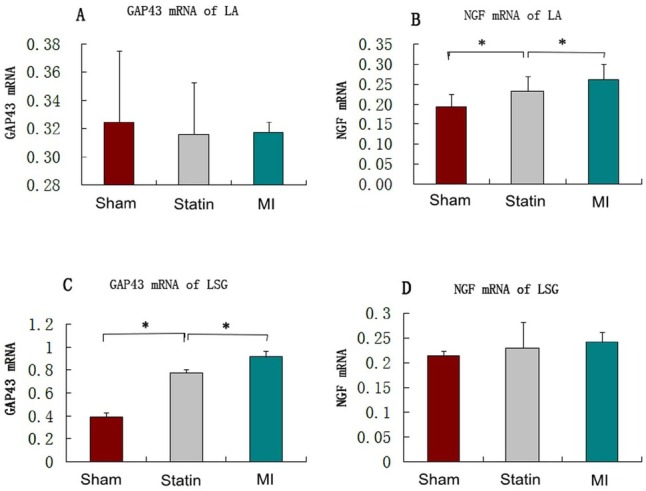
qRT-PCR showing mRNA expression in the LSG and the LA A: Expression levels of GAP43 mRNA in rat LA in the 3 groups; B: Expression levels of NGF mRNA in rat LA in the 3 groups; C: Expression levels of GAP43 mRNA in rat LSG in the 3 groups; D: Expression levels of NGF mRNA in rat LSG in the 3 groups. *: P < 0.01. LA: left atrium; LSG: left stellate ganglion.

The NGF mRNA in the LA were higher in the statin and MI groups than in the sham group (0.231 ± 0.038 and 0.261 ± 0.038 *vs*. 0.192 ± 0.033, *P*<0.01), and this level was lower in the statin group than in the MI group (0.231 ± 0.038 *vs*. 0.261 ± 0.038, *P*<0.01). The mRNA expression levels of GAP43 in the LA among these groups did not exhibit significant differences. The GAP43 mRNA in the LSG increased in both the statin and MI groups (0.762 ± 0.040 and 0.913 ± 0.051 *vs*. 0.385 ± 0.039, *P*<0.01), and this level was lower in the statin group, compared with the MI group (0.762 ± 0.040 *vs*. 0.913 ± 0.051, *P*<0.01). The mRNA expression levels of NGF in the LSG among these groups did not exhibit significant differences (Figure 3).

Protein expression levels of GAP43 and NGF in the LA and LSG in each group after four weeks

**Figure 4:**
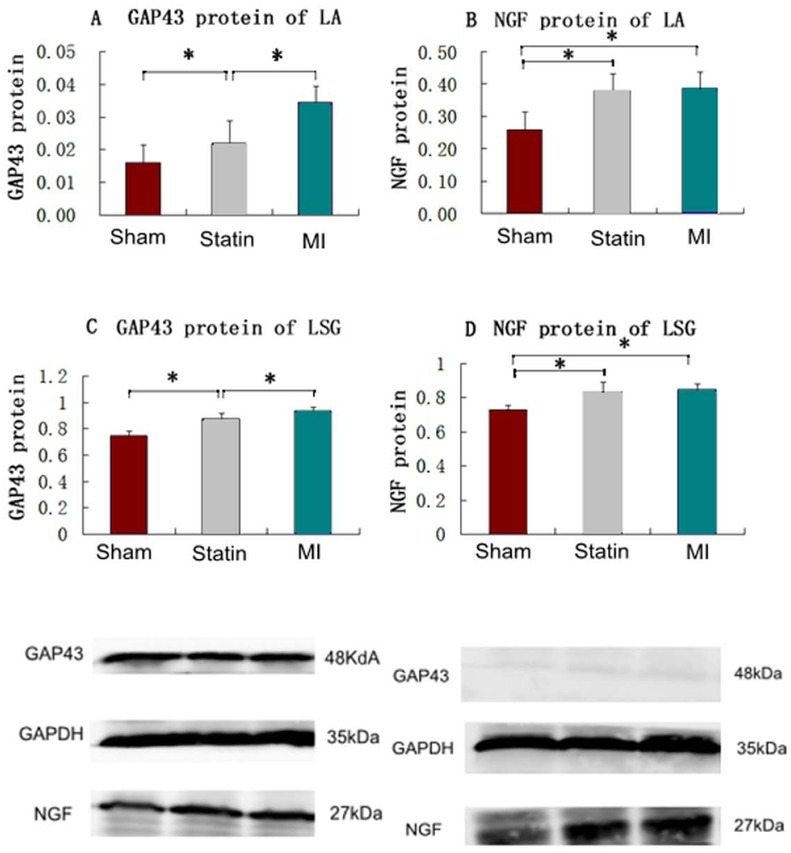
Protein expression of GAP43 and NGF in LA and LSG *Top*: A: Expression of GAP43 protein in rat LA in the 3 groups; B: Expression of NGF protein in rat LA in the 3 groups: C: Expression of GAP43 protein in rat LSG in the 3 groups; D: Expression of NGF protein in rat LSG in the 3 groups. *: P < 0.01. LA: left atrium; LSG: left stellate ganglion. *Bottom*: Sham(LSG): expression of NGF protein in LSG in the sham operation group; Statin(LSG): expression of NGF protein in LSG in the Statin group; MI(LSG): expression of NGF protein in LSG in the MI group. Sham (LA): expression of GAP43 protein in LA in the sham operation group; Statin (LA): expression of GAP43 protein in LA in the Statin group; MI (LA): expression of GAP43 protein in LA in the MI group.

Compared with the sham group, the protein levels of GAP43 in the LA and LSG increased in both the statin (0.022 ± 0.007 *vs*. 0.016 ± 0.005 and 0.870 ± 0.048 *vs*. 0.745 ± 0.036) and MI (0.034 ± 0.005 *vs*. 0.016 ± 0.005 and 0.932 ± 0.029 *vs*. 0.745 ± 0.036) groups (*P*<0.01), and this level of GAP43 was lower in the statin group than in the MI group (0.022 ± 0.007 *vs*. 0.034 ± 0.005 and 0.870 ± 0.048 *vs*. 0.932 ± 0.029, *P*<0.01). Compared with the sham group, the protein levels of NGF in the LA and LSG increased in both the statin (0.379 ± 0.054 *vs*. 0.258 ± 0.056 and 0.848 ± 0.034 *vs*. 0.723 ± 0.029) and MI (0.386 ± 0.051 *vs*. 0.258 ± 0.056 and 0.870 ± 0.048 *vs*. 0.723 ± 0.029) groups (*P*<0.01). The protein levels of NGF in the LA and LSG between the statin and MI groups did not exhibit significant differences (Figure 4).

## 4 Discussion

It has been recently discovered that MI could be accompanied by nerve injury in the heart and SG [8]. Injured nerves could induce repair responses to cause the phenomena of nerve redistribution and hyperinnervation. Sympathetic nerve hyperinnervation could induce a variety of arrhythmias, including AF [3, 4]. The application of rapid right atrial pacing could induce persistent AF in experimental dogs. Furthermore, it has been confirmed that the hearts of these AF animals exhibited a heterogeneous increase in sympathetic nerve innervation in the atria [9], and this increased density could reach 3-5-fold. The mechanism underlying the sympathetic nerve hyperinnervation remains unclear. Some studies have shown that NGF synthesized and released by injured myocardial [10] and inflammatory tissues [11] played important roles in neural remodeling. NGF is one of the important neurotrophic factors in the body. It can promote the development and differentiation of sympathetic nerves and enhance the regeneration and repair of the nervous system after injury. Studies have shown that the level of NGF synthesis in target organs determines the density of nerve regeneration, and its overexpression induces excessive nerve regeneration, thereby causing the phenomenon of neural remodeling [2, 3]. The inhibition of NGF expression decreases the growth of sympathetic nerves in MI tissues [12]. One study suggested that cardiomyocytes synthesized and secreted NGF [13]. NGF could promote the growth of cardiac sympathetic nerves and the regeneration of injured noradrenergic neurons [7]. Therefore, it might participate in the processes of denervation, regeneration and hyperinnervation of sympathetic nerves after MI, thereby being further closely associated with arrhythmia after MI.

GAP43 is a membrane phosphoprotein that is closely associated with neural development and synapse formation and plasticity, particularly nerve regeneration. Certain scholars have considered that NGF could promote GAP43 phosphorylation in growth cones. The sensitivity of PC12 cells transfected with human GAP43 cDNA to NGF increases 10-fold, and the regeneration of neurites significantly increases [14]. After peripheral nerve injury, GAP43 levels could increase 20-100-fold [15, 16]. In specific conditions and environments, the regeneration of peripheral nerve axons was closely associated with GAP43. GAP43 has been considered as a marker for the regeneration of peripheral nerves, because the regeneration of peripheral nerves requires the synthesis of several proteins associated with nerve regeneration, and GAP43 is one of the most important ones [17, 18, 19, 20].

The results of the present study revealed that both the LA and LSG exhibited sympathetic neural remodeling after MI. Recent studies [3, 16] also revealed that after simple left ventricular MI, excessive nerve regeneration occurred, and the inducibility of atrial fibrillation increased, accordingly. Furthermore, the present study also revealed that left ventricular MI induced a significant increase in the density of LA nerve fibers and a disordered distribution. A study [2] revealed that MI could induce the increase of NGF and GAP34 protein levels in the left side of the SG, but did not increase NGF mRNA. These results suggest that the increased NGF in the LSG might be produced by myocardial tissues in the infarction area and transported retrogradely by axons. Furthermore, it was found that the relative mRNA and protein expression levels of NGF in rat atria after MI significantly increased, when compared with the levels in the sham group. Certain scholars have considered that NGF might promote GAP43 phosphorylation in growth cones. The sensitivity of PC12 cells transfected with human GAP43 cDNA to NGF increased 10-fold, and the regeneration of neurites significantly increased [12]. The present study investigated the protein expression of NGF and the sympathetic nerve innervation in rat atria after MI, and these results suggest that the NGF protein expressed in cardiomyocytes played important roles in the process of sympathetic neural remodeling after MI.

The present study revealed that the levels of NGF protein and GAP43 mRNA increased in the LSG in the MI group, suggesting that NGF promoted GAP43 mRNA transcription and expression in the LSG. However, NGF mRNA exhibited no difference in the LSGs in the MI and sham groups. The present results are consistent with the results reported by Zhou *et al*. [2], which revealed that NGF protein in the LSG was produced in the ventricles and retrogradely transported by postganglionic fibers. The studies conducted by the investigators also revealed that compared with the MI group, rosuvastatin intervention could inhibit GAP43 mRNA and protein expression in rat LSG after MI. Therefore, atrial neural remodeling could be improved at this stage. In addition, the present study revealed that rosuvastatin inhibited NGF mRNA expression in rat LSG after MI, but could not decrease the level of NGF protein, suggesting that rosuvastatin cannot inhibit sympathetic neural remodeling in the SG in that pathway. This also further proves that some NGF in rat LSG might come from outside the LSG, having been retrogradely transported by axons. The present study revealed that the level of GAP43 protein detected in rat atria after MI was significantly higher than that in the sham operation group. However, GAP43 mRNA did not exhibit significant differences. It is possible that GAP43 produced by the LSG might be anterogradely transported to the atrium to promote the hyperinnervation of sympathetic nerves in the atrium after MI.

The present study confirms that as the nerves of the LSG are extensively distributed in the whole heart, increased GAP43 protein can promote sympathetic nerve regeneration throughout the heart. The present study also revealed that after rosuvastatin intervention, the protein level of GAP43 decreased in rat LA after MI, but the mRNA level did not change. This result benefited from the inhibitory function of rosuvastatin on the synthesis of GAP43 in the LSG. Rosuvastatin inhibited the expression of NGF mRNA in rat LA after MI, but did not change the NGF protein expression in the LA. One possible reason is that rosuvastatin cannot inhibit an upstream event, such as the expression of ventricular NGF protein and the delivery of NGF protein to the atrium through axonal transport *via* the sympathetic postganglionic fiber.

### 4.1 Limitations

The process of atrial sympathetic nerve regeneration after MI is highly complex. The positive GAP43 expression only suggests the regeneration responses of nerves. This does not necessarily mean a successful regeneration. Furthermore, the lack of GAP43 expression does not lead to the complete inhibition of axonal growth. In addition, the features of GAP43, the relationship between GAP43 and NGF, and the determination on how atrial sympathetic neural remodeling after MI is promoted remain to be further studied.

## 5 Conclusion

Rosuvastatin prevents atrial tachyarrhythmias by improving atrial neural remodeling in rats after MI. Its mechanism may be associated with the downregulation of NGF and GAP43 in the LSG and atrium. These results provide further theoretical basis for the clinical application of statins in MI.
